# The Association of Balance, Fear of Falling, and Daily Activities With Drug Phases and Severity of Disease in Patients With Parkinson

**DOI:** 10.32598/bcn.9.10.295

**Published:** 2019-07-01

**Authors:** Maryam Mehdizadeh, Pablo Martinez-Martin, Seyed Amirhasan Habibi, Negar Nikbakht, Faeze Alvandi, Parvane Bazipoor, Ailin Panahi, Ghorban Taghizadeh

**Affiliations:** 1. Cellular and Molecular Research Center, Iran University of Medical Sciences, Tehran, Iran.; 2. Department of Neuroscience, School of Advance Technologies in Medicine, Iran University of Medical Sciences, Tehran, Iran.; 3. National Center of Epidemiology and CIBERNED, Carlos III Institute of Health, Madrid, Spain.; 4. Department of Neurology, School of Medicine, Rasoul Akram Hospital, Iran University of Medical Sciences, Tehran, Iran.; 5. Department of Occupational Therapy, School of Rehabilitation, Shahid Beheshti University of Medical Sciences and Health Services, Tehran, Iran.; 6. Department of Occupational Therapy, School of Paramedical and Health, Zanjan University of Medical Sciences, Zanjan, Iran.; 7. Department of Pathology and Sport Biomechanics, Faculty of Literature and Humanities, Bu-Ali Sina University, Hamadan, Iran.; 8. Department of Occupational Therapy, School of Rehabilitation Sciences, Iran University of Medical Sciences, Tehran, Iran.; 9. Rehabilitation Research Center, School of Rehabilitation Sciences, Iran University of Medical Sciences, Tehran, Iran.

**Keywords:** Postural balance, Fear, Activities of daily living, Parkinson disease

## Abstract

**Introduction:**

In the elderly, functional balance, fear of falling, and independence in daily living activities are interrelated; however, this relationship may change under the influence of drug phase and the severity of disease in individuals with idiopathic Parkinson disease. This study aimed to investigate the association of functional balance, fear of falling, and independence in the Activities of Daily Living (ADL) with the drug on- and drug off-phases.

**Methods:**

A total of 140 patients with Parkinson disease (age: Mean±SD; 60.51±12.32 y) were evaluated in terms of their functional balance, fear of falling, and independence in their daily activities by the Berg Balance Scale (BBS), Fall Efficacy Scale-International (FES-I), and Unified Parkinson Disease Rating Scale-ADL (UPDRS-ADL), respectively, in drug on- and drug off-phases. The Hoehn and Yahr scale recorded global disease rating. The Spearman coefficient, Kruskal-Wallis, and Mann-Whitney tests were used to find out whether the distribution of scale scores differs with regard to functional balance or disease severity.

**Results:**

A strong correlation was found between the functional balance, fear of falling, and independence in ADL with both drug phases. The results also showed the significant difference in the distribution of the FES-I and UPDRS-ADL scores with regard to functional balance (except independence in ADL in drug off-phase). Also, the distribution of the scores of BBS, FES-I, and UPDRS-ADL showed significant differences with regard to disease severity.

**Conclusion:**

The study showed a strong correlation between functional balance, fear of falling, and independence in ADL that can be affected by the drug phase and severity of the disease. However, more studies are needed to understand this relationship precisely.

## Highlights

- Drug phases may significantly affect different abilities and functions in patients with Parkinson disease.
- Adopting a specific strategy in drug on- and drug off-phases enhances physical functions of these patients.
- It is crucial to choose the best time (drug on- or off-phase) for the examination of changes in different functions.

## Plain Language Summary

Balance impairment and the subsequent increasing fear of falling are two main problems in patients with Parkinson disease that can affect their level of independence in daily activities. The prolonged use of levodopa in these patients results in motor fluctuation, which disturbs various physical functions. Therapists should evaluate people with Parkinson disease in several aspects. First, the level of the disease severity and its impact on functional abilities should be examined. Second, therapists should be aware of drug responses. In this study, the effects of motor fluctuation in two drug phases (on- and off-phase) and severity of the disease were examined on balance, fear of falling, and independence in activities of daily living. The results showed significant relationships between patients’ physical functions and drug phases and disease severity. Considering these associations can lead to better planning of the assessment timing and the therapeutic strategy concerning the drug phase and the severity of the disease.

## 1. Introduction

Parkinson Disease (PD) is a neurodegenerative disorder. One of its main symptoms is the inability to maintain posture and balance (Herman, Weiss, Brozgol, Giladi, & Hausdorff, 2014), a disturbance potentially associated with falling and Fear of Falling (FOF) (Adkin, Frank, & Jog, 2003). FOF is defined as a concern of falling during performing everyday life activities, which may prevent an individual from carrying out his or her activities (Lindholm, Hagell, Hansson, & Nilsson, 2014). According to previous studies in patients with PD, the decreased daily activities due to falling fear is considerable (about 44.1%) (Bloem et al., 2001). Avoiding daily activities because of FOF can increase dependence on others and social isolation while reducing the physical activity and quality of life (Deshpande et al., 2008; Kader, Iwarsson, Odin, & Nilsson, 2016; Rosqvist et al., 2017).

Levodopa is the primary treatment of PD and can be beneficial for almost all patients with PD. As the disease progresses, continuous use of the drug can lead to a series of motor complications (i.e. motor fluctuations). These people experience motor fluctuations between two drug phases called “on” (after taking the first dose of medication while symptoms of the disease are being controlled) and “off” (when symptoms re-emerge, and the patient shows problems in motor function) (Ahlskog, & Muenter, 2001; Rodríguez-Molinero et al., 2017). Previous studies have examined the effects of motor fluctuations on various performances (such as balance, falling, etc.) in both drug phases. The best treatment response, medical interventions, and rehabilitation programs must vary according to the patient’s condition patient’s motor performance and drug phase. Hence, understanding the relationship between factors such as FOF, functional balance abilities, and independence in Activities of Daily Living (ADL) could be useful for planning the most appropriate therapeutic interventions for these patients (Morris, Iansek, & Churchyard, 1998; Morris, Morris, & Iansek, 2001).

However, the relationship between these factors (i.e. functional balance, FOF, and independence in ADL) has not been investigated concerning the severity of the PD. Also, most studies in this area have been conducted in the drug on-phase period. However, functional balance, FOF, and independence in ADL may be significantly different in drug off-phase compared with drug on-phase (Foreman, Addison, Kim, & Dibble, 2011; Franchignoni, Martignoni, Ferriero, & Pasetti, 2005). This study aims to investigate the association of functional balance, FOF, and independence in ADL in people with idiopathic PD with drug on- and drug off-phase.

## 2. Methods

### 2.1. Study participants

The study included 140 subjects with idiopathic PD (age: Mean±SD; 60.51±12.32 y; 66.42% male). The inclusion criteria were having idiopathic PD diagnosed by a neurologist based on UK Parkinson Disease Society Brain Bank clinical diagnostic criteria for PD (Hughes, Daniel, Kilford, & Lees, 1992), lacking any cognitive impairment by obtaining score of >21 on mini-mental status examination (Godefroy et al., 2011), and understanding test instructions in Persian language. All patients with a history of receiving rehabilitation services were excluded from the study.

This study was approved by the Ethics Committee of the Student Research Center at Iran University of Medical Sciences. The participants signed informed consent before participating in this study.

### 2.2. Study procedure

At the beginning of the study, the patients were asked not to use levodopa before coming to the clinic. Assessments were first performed in the drug off-phase (i.e. 12 hours after the last dose of levodopa and before the morning dose) and later in the drug on-phase (i.e. 1 hour after taking the first dose of levodopa in the morning) (Morris et al., 2001). Demographic information (age, sex, duration of disease, the severity of disease based on the Hoehn and Yahr scale, type of drugs, etc.) was obtained using a demographic questionnaire. Functional balance, FOF, and independence in ADL were evaluated using the Berg Balance Scale (BBS), Fall Efficacy Scale-International (FES-I), and Unified Parkinson Disease Rating Scale-ADL (UPDRS-ADL), respectively. The duration of evaluations varied between 30 and 60 minutes (depending on duration of the drug effect).

### 2.3. Study tools

#### 2.3.1. Hoehn and Yahr (HY) Scale

This scale was used to determine the level of PD progression. Based on this scale, the severity of the disease is divided into 5 levels, where level 1 indicates the patient’s normal condition and level 5 indicates patient’s use of a wheelchair (Goetz et al., 2004).

#### 2.3.2. Berg Balance Scale (BBS)

This scale is a basic test for evaluating functional balance and consists of 14 items; each of these items is scored from 0 to 4. The total BBS score ranges from 0 to 56, and the higher score indicates a better balance. BBS evaluates two key dimensions of balance in everyday activities; dynamic and static. The instruments used include chronometer, ruler, chair, and a step. The Persian version of this scale has high inter-rater reliability (ICC=0.99) and internal consistency (the Cronbach α=0.92) in patients with PD (Babaei-Ghazani et al., 2017).

#### 2.3.4. Fall Efficacy Scale-International (FES-I)

This scale has 16 items that measure the FOF. The total score of FES-I ranges between 0 and 64, with a higher score indicating more FOF. The Persian version of this scale has good test-retest reliability (Pearson correlation coefficient=0.70) in the elderly population (Khajavi, 2013).

#### 2.3.5. Unified Parkinson Disease Rating Scale– Activity of Daily Living (UPDRS–ADL)

The second subset of the UPDRS scale (i.e. UPDRS-ADL) was used to measure individuals’ ability and independence in ADL, which contains 13 items (total score range: 0–52). A higher score indicates more dependency on others to carry out daily activities (Shulman et al., 2016).

### 2.4. Statistical analyses

Normality of data was explored by the Kolmogorov-Smirnov test, which indicates the non-normal distribution of data. The Spearman correlation coefficient was used to examine the relationship between functional balance (i.e. BBS score), FOF (i.e. FES-I score), and independence in ADL (i.e. UPDRS-ADL score). The Spearman rank correlation coefficient values of more than 0.60, 0.30–0.59, and less than 0.30 indicate strong, moderate, and weak correlations, respectively (Xie et al., 2006).

Kruskal-Wallis H was investigated between the patients’ FOF and UPDRS-ADL score considering functional balance, which was divided into three levels using BBS score as follows: scores from 56 to 41, level 1; 40 to 21, level 2; less than 20, level 3 (Schneider et al., 2012).

Mann-Whitney test was used to examine the differences in functional balance, FOF, and independence in ADL with regard to PD severity. Different levels of PD severity were determined according to HY stages (stages 1 and 2 or mild were considered as level 1; and stages 3 and 4, or moderate/severe were considered as level 2). Statistical analyses were performed separately for both drug phases (on and off).

## 3. Results

Based on HY scale, 94(67.14%) participants were in stage 1, 46(32.85%) in stage 2. The Mean±SD scores of BBS in drug on-phase were 53.14±5.91, 44.25±13.90 at stages 1 and 2 based on HY scale, respectively. Also, the Mean±SD scores of BBS in drug off-phase were 51.67±7.76, 37.84±16.27 at stages 1 and 2 based on HY scale, respectively.

A moderate correlation was found between functional balance and FOF in the drug on-phase (r=−0.61, P<0.001) and the drug off-phase (r=−0.70, P<0.001). Also, the correlation between functional balance and independence in ADL was moderate in drug on-phase (r=−0.52, P<0.001) and drug off-phase (r=−0.62, P<0.001).

Based on the results of Kruskal-Wallis H test, the distribution of the FES-I and UPDRS-ADL scores in drug on-phase (H=27.44 and H=12.80, respectively) and drug off-phase (H=32.69 and H=21.09, respectively) have significant differences in different levels of functional balance. Mean rank FES-I and UPDRS-ADL scores are presented in Table 1 for BBS levels of functional balance in the drug on-phase and dug off-phase. In all Kruskal-Wallis H tests, P values were less than 0.001.

**Table 1.** Mean rank of FOF and ADL among BBS levels of balance in people with idiopathic Parkinson disease (n=140)

**Drug Phase**	**Level of BBS**	**FOF (FES-I)**	**ADL (UPDRS-ADL)**
On-phase	1	52.59	58.63
	2	94.13	88.35
	3	111.50	100.08
Off-phase	1	51.50	55.72
	2	82.14	80.56
	3	99.00	76.42

BBS. Berg Balance Scale; FOF. Fear of Fall; FES-I. Fall Efficacy Scale-International; ADL. Activities of Daily Living; UPDRS-ADL. Unified Parkinson Disease Rating-Activities of Daily Living.

The results of Man Whitney indicated that the distribution of BBS, FES-I, and UPDRS-ADL scores in drug on- and off-phase were significantly different among HY levels of PD severity. Table 2 presents the mean rank scores of BBS, FES-I, and UPDRS-ADL in the drug on-phase and drug off-phase at varying levels of PD severity. In all tests, P values were less than 0.001.

**Table 2.** Mean rank of balance, FOF and ADL among H-Y levels in people with idiopathic Parkinson’ disease (n=140)

**Level of H-Y**	**Balance (BBS)**		**FOF (FES-I)**		**ADL (UPDRS-ADL)**	

	**Drug on-Phase**	**Drug off-Phase**	**Drug on-Phase**	**Drug off-Phase**	**Drug on-Phase**	**Drug off-Phase**
1	73.86	56.80	52.89	49.69	52.45	51.31
2	43.90	28.18	83.45	82.95	76.60	86.95

H-Y. Hoehn and Yarh; BBS. Berg Balance Scale; FOF. Fear of Fall; FES-I. Fall Efficacy Scale-International; ADL. Activities of Daily Living; UPDRS-ADL. Unified Parkinson Disease Rating-Activities of Daily Living.

## 4. Discussion

The purpose of this study was to investigate the association of functional balance, FOF, and independence in ADL in people with idiopathic PD with drug on-phase and drug off-phase. The results of this study showed a significant difference between various levels of functional balance and severity of the disease, as well as FOF and independence in ADL in both drug on- and drug offphases.

According to studies of Franchignoni et al. (2005), Bryant, Rintala, Hou, & Prota (2015), and Landers et al. (2017), people with greater damage in balance show more FOF during their functions (Bryant et al., 2015; Franchignoni et al., 2005; Landers et al., 2017). Also according to results of Landers et al. (2017) study, FOF is significantly different at varying stages of disease severity (Landers et al., 2017), which is consistent with the results of our research. Also, as suggested by Foreman et al. (2011) who used a correlation analysis, evaluation of FOF and independence in ADL at different levels of functional balance in drug off-phase gives more accurate information to therapists in these cases (Foreman et al., 2011). On the other hand, results at different levels of functional balance showed a significant change in independence in ADL in drug on-phase and drug off-phase. This outcome in our study may be due to a decrease in physical activity, as a result of a reduction in the effect of dopamine medications in the brain, which needs further investigation.

One of the important factors for independence in ADL, especially in those needing a standing position, is to maintain the balance and to control the posture. Landers et al. (2017) found that patients with PD avoid doing such activities because of the lack of functional balance to prevent falling (Landers et al., 2017). The results of this study also showed that at better levels of functional balance, independence in ADL was higher. These results were more pronounced in drug off-phase, which alters the functional state of the patients with PD.

Finally, the findings of this study indicate that taking a plan (rehabilitation and medication) to improve the balance of people with idiopathic PD can help reduce FOF. It is noteworthy that when individuals are less worried about falling during their ADL, their level of independence will become relatively higher.

Additionally, increasing dependence on others due to the reduction of independence in ADL can increase the feeling of tiredness and burden among the caregivers. Since PD is a chronic and progressive disorder, interventions designed to improve the level of independence in ADL can indirectly help the caregivers (Bhatia et al., 2003).

Maintaining balance and posture in a standing position is essential for most of the daily activities (Dunsky, Zeev, & Netz, 2017). According to the results of this study, in people with more balance problem, the fear of falling and dependence on others were higher. This case was shown in this study with the progression of the disease as well as in the off-phase drug.

We believe that consideration of balance training in the rehabilitation program of these individuals can help maintain independence in everyday activities, even at advanced levels of disease and in the drug off-phase (in which symptoms of disease reappear). Of course, more comprehensive studies are needed to confirm this conclusion.

This study has some limitations. First, psychological factors were not considered in this study. Moreover, individuals were selected with a convenience sampling method, and, thus, most of the samples belonged to stage 1 of PD severity. These limitations may reduce the degree of generalizability of the results. Hence, it is suggested that future studies consider these issues.

In conclusion, considering the association of functional balance, FOF, and independence in ADL in patients with PD with drug on-off phase, it is critical to design an effective treatment plan. Identification and management of difficulties in balance and ADL, as well as FOF, may increase the quality of life and the level of participation of individuals in social activities (regardless of the severity of disease and drug phases).
